# Chronic Administration of Hydroxyurea (HU) Benefits Caucasian Patients with Sickle-Beta Thalassemia

**DOI:** 10.3390/ijms19030681

**Published:** 2018-02-28

**Authors:** Rosario Di Maggio, Matthew M. Hsieh, Xiongce Zhao, Giuseppina Calvaruso, Paolo Rigano, Disma Renda, John F. Tisdale, Aurelio Maggio

**Affiliations:** 1 Campus of Haematology Franco and Piera Cutino, AOOR Villa Sofia-V. Cervello, 90142 Palermo, Italy; rdm83@hotmail.it (R.D.M.); calvaruso.giuseppina@gmail.com (G.C.); paolorigano@tiscali.it (P.R.); dismare@gmail.com (D.R.); 2 Molecular and Clinical Hematology Branch, National Institute of Diabetes and Digestive and Kidney Diseases/National Heart, Lung, and Blood Institute, Bethesda, MD 20814, USA; matthewhs@nhlbi.nih.gov (M.M.H.); johntis@nhlbi.nih.gov (J.F.T.); 3 Office of Clinical Director, National Institute of Diabetes and Digestive and Kidney Diseases, Bethesda, MD 20814, USA; Xiongce.Zhao@fda.hhs.gov

**Keywords:** sickle cell disease, sickle beta thalassemia, hydroxyurea, fetal hemoglobin

## Abstract

In sickle cell disease (SCD), hydroxyurea (HU) treatment decreases the number of vaso-occlusive crisis (VOC) and acute chest syndrome (ACS) by increasing fetal hemoglobin (HbF). Data are lacking regarding the frequency of HU dose modification or whether sub-therapeutic doses (<15 mg/kg/day) are beneficial. We reviewed the medical records of 140 patients from 2010 to 2014. The laboratory parameters and SCD complications were compared between the first and last visits based on HU use. Fifty patients (36%) never took HU or suspended HU (“no HU” group). Among patients taking <15 mg/kg/day HU on their first visit, half remained at the same dose, and the other half increased to ≥15 mg/kg/day. Among patients taking ≥15 mg/kg/day, 17% decreased to <15 mg/kg/day, and 83% stayed at ≥15 mg/kg/day. The “no HU” group had fewer episodes of VOC and ACS. Both HU treatment groups had a reduction in both complications (*p* < 0.0001). This improvement was observed in all SCD phenotypes. The white blood cell (WBC) counts were found to be lower, and HbF increased in both HU groups (*p* = 0.004, 0.001). The maximal HbF response to HU in HbS/β^+^-thalassemia was 20%, similar to those observed for HbSS (19%) and HbS/β^0^-thalassemia (22%). HbS/β^+^-thalassemia could have a similar disease severity as HbSS or HbS/β^0^-thalassemia. Patients with HbS/β^0^-thalassemia or HbS/β^+^-thalassemia phenotypes responded to HU.

## 1. Introduction

Sickle cell disease (SCD) is the most common inherited hemoglobin disorder in Africa and has prompted the World Health Organization (WHO) to call for the implementation of regional strategies to increase disease awareness and provide a national infrastructure to reduce its morbidity and mortality [1]. Overall, 5% of the global population are carriers for hemoglobinopathies, with 40% of them being represented by HbS [2,3]. The burden of this and other hemoglobin disorders is expected to increase in the coming decades because of the reductions in infant mortality in many low-income countries and the increasing migration from high- to low-HbS-frequency areas worldwide [4].

Childhood deaths have declined over the last 3 decades in Europe and North America due to newborn screening, routine prophylaxis with penicillin, pneumococcal vaccinations, and access to medical care [5,6,7,8]. Despite these advances, mortality due to disease and organ dysfunctions has remained high in patients with SCD. The median age of death was reported to be 42 years for men and 48 years for women [9,10]. 

Hydroxyurea (HU) has been approved for treating SCD [11]. HU increases production of fetal hemoglobin (HbF) with a concomitant reduction in the intracellular concentration of HbS, which affects the polymerization of deoxygenated HbS. It also reduces the white blood count (WBC) and the expression of cell adhesion molecules that contribute to vaso-occlusion and may serve as a nitric oxide donor [12,13,14,15]. Long-term studies demonstrated its efficacy in decreasing both the number and severity of vaso-occlusive crisis (VOC) and acute chest syndromes (ACS) by increasing fetal hemoglobin (HbF), leading to a reduction of red cell sickling [16,17,18,19,20]. Although the long-term use of HU has improved overall survival, it was not clear what proportions of patients were taking a therapeutic dose of ≥15 mg/kg/day [21,22]. There was also inadequate reporting of the frequency of HU dose modification during follow-up and whether those who were treated with doses <15 mg/kg/day benefitted from HU. In this work, we evaluated the effects of different HU doses in patients with SCD in terms of hematologic improvement, possible changes in organ dysfunction, and causes of death at a single referral institution. 

## 2. Results

### 2.1. Hydroxyurea Dosing Adjustment Was Common

The 140 patients at the Hospital “V. Cervello” had a median enrollment age of 35 years (range 0.4–61 years) with equal gender proportions (Table 1). The mean duration of follow-up was 6.6 years. The criteria to start HU treatment were those suggested by Charache et al., 1995 (>3 painful vaso-occlusive crises per year and/or >2 Acute Chest Syndrome [20]). HU was started at a dose of 10 mg/kg orally, on a daily basis, and increased at a rate of 5 mg/kg/week as long as the hematologic values remained in an acceptable range. Among 64 patients who did not take HU at the initial visit, 39 stayed off treatment, 10 patients started HU later and maintained the dose at <15 mg/kg/day, and another 15 patients started and maintained HU at ≥15 mg/kg/day at the last visit. 

Of 76 patients who were taking HU at the first visit, 11 discontinued HU by the last clinic visit due to cytopenia: 7 patients had a reduction in neutrophil counts, 3 patients suffered decreased hemoglobin levels, and 1 patient had a decrease in platelet counts. These 11 patients, combined with the 39 patients who never took HU, formed the “no HU” group (*n* = 50 of 140, 36%). Among patients who took <15 mg/kg/day HU at the first visit, 14 stayed in the same dosage, and 15 increased to ≥15 mg/kg/day at the last follow-up. Of the patients who took ≥15 mg/kg/day, 6 decreased to <15 mg/kg/day due to neutropenia, and 30 stayed in the same dose range. Overall, there were 30 patients (21%) in the “HU <15 mg/kg/day” group and 60 (43%) in the “HU ≥ 15 mg/kg” group. 

### 2.2. Hydroxyurea Led to Similar Beneficial Hematologic Changes in All Sickle Phenotypes

Patients with HbSS (18%) represented a small proportion of all patients compared to HbS/β^0^ (39%) and combined HbS/β^+^ (43%). The median age, proportion of males and females, and survival status were similar in all three groups (Table 2). The proportion of patients taking HU, increases in HbF, and increases in MCV, were also similar. 

At the last visit, the mean white blood cell (WBC) count was lower in both HU groups compared to the No-HU group (*p* < 0.05) (Supplemental Table S2). The mean absolute neutrophil count (ANC) and total hemoglobin levels in both HU groups, however, were not significantly different than the No-HU group, possibly due to HU not being at the maximally tolerated doses. 

The HbF levels in the No-HU group at the first visit were higher due to the inclusion of four patients with high HbF levels and two < one-year-old children. The HbF levels in this group subsequently decreased at the last visit. In contrast, both HU groups had increased HbF levels at the last visit, and these changes were significant (*p* < 0.05) compared with the No-HU group (Supplemental Table S2). HU-treated groups showed HbF last visit/HbF first visit ratios >1. These ratios were 1.12 and 1.24 in the ≥15 mg/kg/day and <15 mg/kg/day HU-treated groups, respectively. Instead, No HU-treated patients showed HbF last visit/HbF first visit ratio <1 (ratio 0.65). 

However, HU treatment was not associated with changes in liver enzymes, serum creatinine (sCr), tricuspid valve regurgitant velocity (TRV), or ejection fraction (EF) (Supplemental Table S2). 

### 2.3. Hydroxyurea Reduced VOC and ACS Events

The overall 50 patients in the No HU-group, compared to both HU groups at the first visit, had milder disease with lower percentage of patients with VOC (60% vs. 94%) and ACS (22% vs. 50.5%) (*p* = 0.001). 

The prevalence of patients having ≥1 new VOC hospitalization or ≥1 new ACS at first and last visit was similar (49% vs. 67% for VOC and 18% vs. 30% for ACS), if the 39/50 No-HU sub-group of patients who never took HU was considered. These findings suggest as the inclusion, in the No-HU treated group, of the cohort with 11 patients who discontinued drug treatment before last visit, may not have any role in increasing complications. 

The No-HU group follow-up suggests as the prevalence of patients with ≥1 new VOC hospitalization before the last visit was 70%, very close to the 60% at the first visit. 

The number of VOC episodes per patient /year was even similar (2.0 and 2.5 at first and last visit, respectively). 

ACS prevalence, in the No-HU treated group, was similar between the first (22%) and the last visit (28%), respectively. Finally, even the number of mean episodes of ACS per patient was similar (0.2 vs. 0.28) 

In contrast, both HU-treated groups, <15 mg/kg/day and ≥15 mg/kg/day, had higher prevalence of VOC at the first (90%) and last visit (97%), respectively. However, these values decreased significantly at the last visit, moving from 90 to 57% and from 97 to 60%, respectively. 

The number of VOC episodes per patient/year decreased from 4.3 to 1.2 and from 4.1 to 1.1, in the HU group <15 mg/kg/day, and in the HU-group ≥15 mg/kg/day, respectively.

The number of mean ACS episodes per patient in both HU groups also decreased by nearly 2/3, from 0.7 to 0.23 and from 1.1 to 0.32, respectively. 

**Figure 1:**
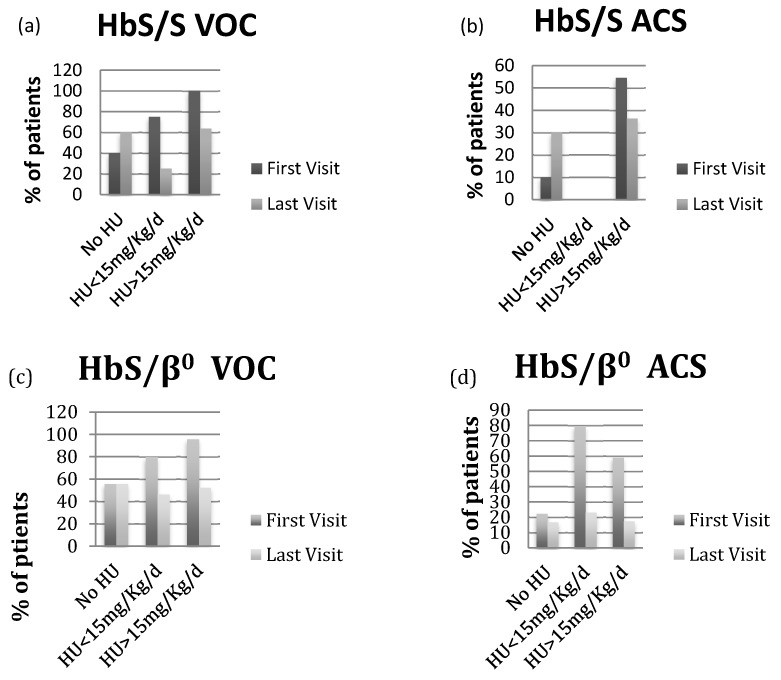

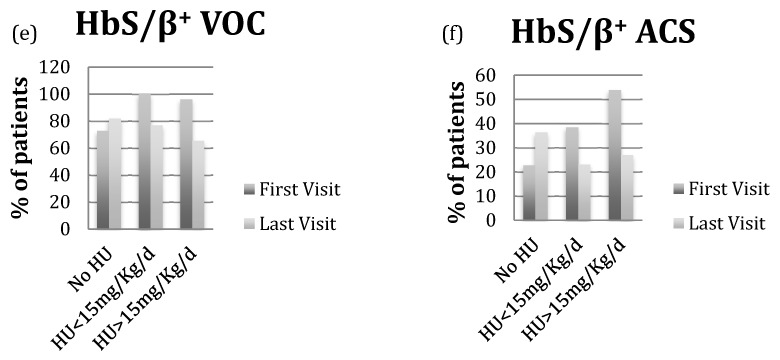
(**a**,**c**,**e**) VOC prevalence at first and last visit based on HU status in patients with HbS/S, HbS/β^0^-thalassemia and HbS/β^+^-thalassemia; (**b**,**d**,**f**) ACS prevalence at first and last visit based on HU status in patients with HbS/S, HbS/β^0^-thalassemia and HbS/β^+^-thalassemia.

The patients with HbS/β^+^-thalassemia had also severe phenotype, when they were compared to others. Indeed, the same proportion of patients with HbS/β^0^- and HbS/β^+^-thalassemia died during the follow-up period (Table 2). Additionally, the prevalence of patients with VOC and ACS, the number of VOC per patient/year, the number of ACS episodes per patient were similar among the three phenotypes. Finally, at the last visit, all three phenotype groups had the similar reduction in VOC and ACS on HU treatment (Figure 1). 

### 2.4. Mortality

Fifteen deaths have been registered over 6.6 years follow-up. The median age of death was 46 years (24–68 years), with a male to female ratio of 2:1. Deceased patients at the first visit were significantly older (42.06 vs. 33.13 years). Among the 15 deaths, five patients did not received treatment with HU, four patients received HU at <15 mg/kg/day and 5 patients at ≥15 mg/kg/day; one patient took HU <15 mg/kg/day but it was discontinued at the last visit. Worst hepatic, renal, cardiopulmonary, and iron overloading findings at the first visit were shown in patients who died in comparison with those who did not; the same pattern was observed at the last visit (Table 3).

There were no differences in hematologic parameters between the alive and deceased patients at the first visit (Hb 9.9 vs. 10.0 g/dL, MCV 82 vs. 81 fL, WBC 8.9 vs. 10.4 K/µL, HbF 12.2 vs. 12.1%). However, significant differences were observed at the last follow-up (dead vs. alive, Hb 9.4 vs. 11.3 g/dL, MCV 82 vs. 85 fL, WBC 12.6 vs. 8.4 K/µL, HbF 11.5 vs. 11.8%). Overall causes of death may be considered sickle-cell related: 4 deaths for ACS, 1 for Acute Respiratory Distress Syndrome (ARDS) 1 for VOC, 1 for sepsis, 2 for post-ischemic intestinal hemorrhage, during VOC, 3 for myocardial infarction and 3 for cirrhosis with liver failure in HCV-RNA negative patients.

## 3. Discussion

SCD, even under contemporary medical care, is burdened by high morbidity and mortality [23]. The introduction of HU, the only medication approved for its treatment, has improved the outcome of many patients with SCD [11]. 

However, since its approval, it is not clear which proportion of patients is taking a therapeutical dosage (≥15 mg/kg/day) and/or the rate at which HU dosage changes occur during the follow-up.

These findings suggest as, in our adult cohort of patients with Caucasian Sickle-Cell-Beta Thalassemia, during an average follow-up of 6.6 years, HU was administered to 54% and to 64% of patients at the initial and final visit, respectively. Moreover, the dosage adjustment was common.

A large proportion of No HU-treated patients at the initial visit (25 out of 64, 39%) began and remained on HU by the last visit. An equally large proportion of HU <15 mg/kg/day patients at the initial visit (15 out of 29, 52%) increased HU dosage to ≥15 mg/kg/day. The HU ≥ 15 mg/kg/day dosing group appeared to be the most stable, with 30 out of the 36 patients (86%) remaining above 15 mg/kg/day. 

Most of dose reductions in HU group were due to neutropenia during HU escalation dosage aimed at reaching the maximally tolerated dose.

The rate of HU administration, in this Caucasian cohort of patients with Sickle-Cell Beta-Thalassemia, was comparable with the recent papers reporting as HU rate of administration was 50% both in the inpatient cohort of a multi-center study [24] and in the US federal and state insurance-based analysis [25]. While it was prescribed from 50 to 75% by US hematologists [26,27]. 

The main hematological findings between first and last visit suggest as: (1) WBC counts were lower in HU-treated groups; (2) hemoglobin levels changing were not statistically significant during the observation period; (3) the HbF levels at the last visit increased significantly in HU-treated groups compared with No HU-treated group (*p* < 0.05).

These data confirm as even in Caucasian population with Sickle-Cell/ beta-thalassemia, HU is able to decrease WBC count and to increase the HbF, percentage [20,21,22,23,24,25,26,27,28,29,30]. These changes are associated with decreasing of VOC, ACS, and the other complications [30,31,32]. Recently, Rigano et al., in a cohort of 652 Sickle-Cell Anemia adult patients with heterogeneous descent, suggested as HU treatment was associated with a significant increase in mean total and fetal hemoglobin and a significant decrease in mean white blood and platelet count [32].

This study confirmed the data about mortality that were shown in prior studies over the last several years—pulmonary and cardiac complications, with a vaso-occlusive crisis are the most common causes of death in patients with SCD [27,33,34]. In our study, ACS after VOC was a more common cause of death, followed by heart failure during a VOC. 

Our study showed that patients HbSS, HbS/β^0^-thal, and HbS/β^+^-thal all responded to HU similarly. Patients in each subgroup showed improved hematological parameters (WBC, ANC, MCV, HbF) after the treatment, suggesting that higher dosages led to better results. Unfortunately, the small sample size precluded subtype analysis.

The limitations of this retrospective study were due to the lack of patient follow-up data from birth. Moreover, HU treatment decision and doses were made at the discretion of the treating physician. Furthermore, compliance with HU was inferred by medical record reviews and not independently verified, so HU use may have been intermittent.

However, these data suggest, as in real-life and in a Caucasian population with Sickle-Cell Beta Thalassemia, HU treatment is effective and safe at long-term.

## 4. Methods

### 4.1. Patient Population

Medical records were retrospectively reviewed, and 140 patients were enrolled at the Haematology Department of Ospedale V. Cervello between January 2000 and April 2014. SCD subtypes were determined by DNA sequencing and high-performance liquid chromatography (HPLC): 25 patients (18%) were homozygous for hemoglobin S (HbSS); 54 (38%) with compound heterozygous for HbS/β^0^-thalassemia; 56 with HbS/β^+^-thalassemia, 4 with HbS/δβ-thalassemia, and 1 HbS/Lepore (44%, Supplemental Table S1).

The patients were also divided into three groups based on HU use: patients who never took HU and those who suspended HU intake before the last visit (No HU group, *n* = 50, 36%); those who reported taking HU at <15 mg/kg/day at the last visit (*n* = 30, 21%); or those taking HU ≥15 mg/kg/day at the last visit (*n* = 60, 43%). 

### 4.2. Clinical, Laboratory, and Echocardiographic Data 

Clinical, laboratory, and echocardiographic evaluations were performed at the first visit and every three to six months during follow-up, when appropriate. Data analysis included inpatient and outpatient visits to Hospital “V. Cervello” following enrollment. Data obtained from outside hospitals close to the time of death were included for comparison. For each subject, the maximum HbF, mean corpuscular volume (MCV), and maximum HU dose were defined as the highest values observed during the follow-up. The frequency of VOC and ACS episodes at the first visit were compared to new reports of these sickle-related complications since the first visit. Blood counts, HbF response, organ function parameters, and changes in SCD complications were compared between the first and last visits. 

### 4.3. Hydroxyurea Status and Dose Determination

HU treatment and dosing decisions were made at the discretion of the treating physician. The dose determinations were screened by electronic outpatient clinic notes, inpatient admission records, and medication orders. 

### 4.4. Statistical Analysis

Groups were analyzed based on survival status, hydroxyurea dosing, and HbF response. Comparisons were performed using *t*-tests, Wilcoxon rank tests, and chi-square tests, when appropriate. A Cox proportional hazard regression was used to relate survival and hydroxyurea exposure with laboratory values and other potential covariates. All analyses were performed using SAS (version 9.1.3; SAS Institute, Cary, NC, USA) and JMP (version 8.0; SAS Institute, Cary, NC, USA). 

## Figures and Tables

**Table 1:** Demographics and medical treatments at the last clinic visit.

Variable		All (*N* = 140)	Alive (*N* = 125)	Deceased (*N* = 15)
Age at enrollment (years)		35 (0.4–61)	35 (0.4–61)	42 (20–60)
Gender	Male	69 (49%)	59 (47%)	10 (67%)
	Female	71 (51%)	66 (53%)	5 (33%)
Hydroxyurea Status	Yes	90 (72%)	81 (73%)	9 (60%)
	No	50 (28%)	44 (27%)	6 (40%)
Transfusion	Yes	48 (34%)	43 (34%)	5 (33%)
	No	92 (66%)	82 (66%)	10 (67%)
Chelation	Yes	40 (29%)	32 (26%)	8 (53%)
	No	100 (71%)	93 (74%)	7 (47%)

**Table 2:** Patient characteristics and hydroxyurea use among sickle phenotypes at last clinic visit.

Variable		HbSS (*N* = 25)		HbS/β^0^ thal (*N* = 54)		HbS/β^+^ thal, δβthal, Lepore (*N* = 61)	
		HU No (*N* = 10)	HU Yes (*N* = 15)	HU No (*N* = 18)	HU Yes (*N* = 36)	HU No (*N* = 22)	HU Yes (*N* = 39)
Age (years)		31.5 (22–44)	32.7 (13–44)	35.3 (0.41–57)	31.9 (5–61)	38 (1–60)	34.4 (7–56)
Gender	Male	4 (40%)	7 (47%)	7 (39%)	20 (55%)	8 (36%)	23 (59%)
	Female	6 (60)	8 (53%)	11 (61%)	16 (45%)	14 (64%)	16 (41%)
Survival Status	Alive	9 (90%)	15 (100%)	16 (89%)	31 (86%)	19 (86%)	35 (90%)
	Deceased	1 (10%)	0 (0%)	1 (11%)	5 (14%)	3 (14%)	4 (10%)
Hydroxyurea Dosage, N (%)	No HU	10 (100%)	0 (0%)	18 (100%)	0 (0%)	22 (100%)	0 (0%)
	<15 mg/kg/d	0 (0%)	4 (16%)	0 (0%)	13 (36%)	0 (0%)	13 (33%)
	≥15 mg/kg/d	0 (0%)	11 (84%)	0 (0%)	23 (64%)	0 (0%)	26 (67%)
Maximum HbF (%)	No HU	16.1 ± 12.2	0	28.7 ± 17.3	0	11.5 ± 10.1	0
	<15 mg/kg/d	0	12.8 ± 8.3	0	25.9 ± 9	0	9 ± 7
	≥15 mg/kg/d	0	19.3 ± 7.8	0	21.6 ± 11.9	0	19.6 ± 7.6
Mean HbF (%)	No HU	11.2 ± 11.8	0	10.1 ± 9.2	0	9.4 ± 9.7	0
	<15 mg/kg/d	0	10.1 ± 4.9	0	8.8 ± 6.3	0	4.7 ± 3.1
	≥15 mg/kg/d	0	10.2 ± 6.9	0	15 ± 11.1	0	10.9 ± 6.8
Maximum MCV	No HU	101 ± 12	0	89 ± 14	0	79 ± 10	0
	<15 mg/kg/d		115 ± 14	0	95 ± 10	0	86 ± 13
	≥15 mg/kg/d		124 ± 18	0	94 ± 12	0	100 ± 9

**Table 3:** Hematologic and Organ Function Parameters in Patients Alive and Deceased at First and Last Visit.

Variable	First Visit		Lst Visit	
	Alive	Deceased	Alive	Deceased
White Blood Count (K/µL)	10.08 ± 1.71	8.9 ± 3.13	10.87 ± 8.65	11.81 ± 9.52
ANC (K/µL)	5.59 ± 3.13	4.73 ± 1.85	4.68 ± 2.24	4.54 ± 2.95
Hemoglobin (g/dL)	10.08 ± 1.71	9.72 ± 1.38	10.87 ± 8.65	9.5 ± 1.21
MCV (fL)	81.12 ± 13.45	82.97 ± 11.21	85.08 ± 13.65	81.82 ± 7.45
Platelet Count (K/µL)	366.72 ± 207.92	299.9 ± 174.84	338.54 ± 192.24	244.33 ± 112.95
Reticolocyte (%)	6.87 ± 4.77	6.38 ± 2.72	6.04 ± 3.60	7.65 ± 3.91
Hemoglobin F (%)	10.7 ± 10.1	11.96 ± 9.43	10.86 ± 8.98	10.68 ± 10.41
Alkaline Phosphatase (U/L)	134.78 ± 96.08	191.93 ± 100.14	77.4 ± 53.71	197.13 ± 140.42
ALT (U/L)	29.04 ± 17.32	53.66 ± 47.19	31.87 ± 20.01	46.46 ± 31.04
AST (U/L)	38.21 ± 21.65	69.8 ± 43.43	38.38 ± 22.25	93.33 ± 63.93
Totl Bilirubin (mg/dL)	2.44 ± 1.90	3.23 ± 2.1	2.2 ± 2.06	7.24 ± 8.9
Direct Bilirubin (mg/dL)	0.41 ± 0.35	0.98 ± 1.03	0.43 ± 0.66	3.14 ± 2.98
Creatinine (mg/dL)	0.65 ± 0.41	0.72 ± 0.26	0.58 ±	0.74 ± 0.33
Ejection Fraction (%)	63.93 ± 5.69	64.1 ± 4.98	62.51 ± 7.46	64.14 ± 6.64
TRV (m/s)	2.49 ± 0.52	2.95 ± 0.36	2.67 ± 0.33	2.8 ± 0.8
Ferritin (mcg/L)	536.89 ± 730.40	1338.8 ± 1485.95	648.65 ± 858.47	1927.96 ± 1654.50
Iron (mcg/L)	109.28 ± 55.67	153.86 ± 79.01	116.76 ± 53.63	160.4 ± 71.23

ANC: Absolute Neutrophil Count; MCV: Mean Corpuscular Volume; AST: Aspartate Aminotransferase; ALT: Alanine Aminotransferase; TRV: Tricuspid Regurgitant Velocity

## Supplementary Materials

Supplementary materials can be found at http://www.mdpi.com/1422-0067/19/3/681/s1.

## References

[1] World Health Organization Regional Office for Africa (2010). Sickle-Cell Disease: A Strategy for the WHO African Region.

[2] Modell B., Darlison M. (2008). Global epidemiology of aemoglobin disorders and derived service indicators. Boll. World Health Organ..

[3] Piel F.B., Patil A.P., Howes R.E., Nyangiri O.A., Gething P.W., Dewi M., Temperley W.H., Williams T.N., Weatherall D.J., Hay S.I. (2013). Global epidemiology of sickle haemoglobin in neonates: A contemporary geostatical model-based map and population estimates. Lancet.

[4] Weatherall D.J. (2010). The inherited diseases of hemoglobin are an emerging global health burden. Blood.

[5] Gaston M.H., Verter J.I., Woods G., Pegelow C., Kelleher J., Presbury G., Zarkowsky H., Vichinsky E., Iyer R., Lobel J.S. (1986). Prophylaxis with oral penicillin in children with sickle cell anemia. N. Engl. J. Med..

[6] Falletta J.M., Woods G.M., Verter J.I., Buchanan G.R., Pegelow C.H., Iyer R.V., Miller S.T., Holbrook C.T., Kinney T.R., Vichinsky E. (1995). Discontinuing penicillin prophylaxis in children with sickle cell anemia. Prophylactic Penicillin Study II. J. Pediatr..

[7] Halasa N.B., Shankar S.M., Talbot T.R., Arbogast P.G., Mitchel E.F., Wang W.C., Schaffner W., Craig A.S., Griffin M.R. (2007). Incidence of invasive pneumococcal disease among individuals with sickle cell disease before and after the introduction of the pneumococcal conjugate vaccine. Clin. Infect. Dis..

[8] McGann P.T., Nero A.C., Ware R.E. (2013). Current Management of Sickle Cell Anemia. Cold Spring Harb. Perspect. Med..

[9] National Institutes of Health NHLBI (2002). The Management of Sickle Cell Disease.

[10] Hamideh D., Alvarez O. (2013). Sickle cell disease related mortality in the United States (1999–2009). Pediatr. Blood Cancer.

[11] Elmariah H., Garrett M.E., De Castro L.M., Jonassaint J.C., Ataga K.I., Eckman J.R., Ashley-Koch A.E., Telen M.J. (2014). Factors associated with survival in a contemporary adult sickle cell disease cohort. Am. J. Hematol..

[12] Charache S., Barton F.B., Moore R.D., Terrin M.L., Steinberg M.H., Dover G.J., Ballas S.K., McMahon R.P., Castro O., Orringer E.P. (1996). Hydroxyurea and sickle cell anemia: Clinical utility of a myelosuppressive “switching” agent. The Multicenter Study of Hydroxyurea in Sickle Cell Anemia. Medicine (Baltim.).

[13] Kato G.J., Gladwin M.T., Steinberg M.H. (2007). Deconstructing sickle cell disease: Reappraisal of the role of hemolysis in the development of clinical subphenotypes. Blood Rev..

[14] Benkerrou M., Delarche C., Brahimi L., Fay M., Vilmer E., Elion J., Gougerot-Pocidalo M.A., Elbim C. (2002). Hydroxyurea corrects the dysregulated l-selectin expression and increased h2o2 production of polymorphonuclear neutrophils from patients with sickle cell anemia. Blood.

[15] Gladwin M.T., Shelhamer J.H., Ognibene F.P., Pease-Fye M.E., Nichols J.S., Link B., Patel D.B., Jankowski M.A., Pannell L.K., Schechter A.N. (2002). Nitric oxide donor properties of hydroxyurea in patients with sickle cell disease. Br. J. Haematol..

[16] Ware R.E., Aygun B. (2009). Advances in the use of hydroxyurea. Hematol. Am. Soc. Hematol. Educ. Program.

[17] Dover G.J., Charache S. (1992). Hydroxyurea induction of fetal hemoglobin synthesis in sickle-cell disease. Semin. Oncol..

[18] Cokic V.P., Smith R.D., Beleslin-Cokic B.B., Njoroge J.M., Miller J.L., Gladwin M.T., Schechter A.N. (2003). Hydroxyurea induces fetal hemoglobin by the nitric oxide-dependent activation of soluble guanylyl cyclase. J. Clin. Investig..

[19] Ho J.A., Pickens C.V., Gamcsik M.P., Colvin O.M., Ware R.E. (2003). In vitro induction of fetal hemoglobin in human erythroid progenitor cells. Exp. Hematol..

[20] Charache S., Terrin M.L., Moore R.D., Dover G.J., Barton F.B., Eckert S.V., McMahon R.P., Bonds D.R. (1995). Effect of hydroxyurea on the frequency of painful crises in sickle cell anemia. Investigators of the Multicenter Study of Hydroxyurea in Sickle Cell Anemia. N. Engl. J. Med..

[21] Ferster A., Vermylen C., Cornu G., Buyse M., Corazza F., Devalck C., Fondu P., Toppet M., Sariban E. (1996). Hydroxyurea for treatment of severe sickle cell anemia: A pediatric clinical trial. Blood.

[22] Steinberg M.H., McCarthy W.F., Castro O., Ballas S.K., Armstrong F.D., Smith W., Ataga K., Swerdlow P., Kutlar A., DeCastro L. (2010). The risks and benefits of long-term use of hydroxyurea in sickle cell anemia: A 17.5 year follow-up. Am. J. Hematol..

[23] Wang W.C., Ware R.E., Miller S.T., Iyer R.V., Casella J.F., Minniti C.P., Rana S., Thornburg C.D., Rogers Z.R., Kalpatthi R.V. (2011). Hydroxycarbamide in very young children with sickle-cell anaemia: A multicentre, randomised, controlled trial (BABY HUG). Lancet.

[24] Wang W., Brugnara C., Snyder C., Wynn L., Rogers Z., Kalinyak K., Brown C., Qureshi A., Bigelow C., Neumayr L. (2011). The effects of hydroxycarbamide and magnesium on haemoglobin SC disease: Results of the multi-centre CHAMPS trial. Br. J. Haematol..

[25] Voskaridou E., Christoulas D., Bilalis A., Plata E., Varvagiannis K., Stamatopoulos G., Sinopoulou K., Balassopoulou A., Loukopoulos D., Terpos E. (2010). The effect of prolonged administration of hydroxyurea on morbidity and mortality in adult patients with sickle cell syndromes: Results of a 17-year, single-center trial (LASHS). Blood.

[26] Lobo C., Hankins J.S., Moura P., Plata E., Varvagiannis K., Stamatopoulos G., Sinopoulou K., Balassopoulou A., Loukopoulos D., Terpos E. (2010). Hydroxyurea therapy reduces mortality among children with sickle cell disease. ASH Annu. Meet. Abstr..

[27] Platt O.S., Brambilla D.J., Rosse W.F., Milner P.F., Castro O., Steinberg M.H., Klug P.P. (1994). Mortality in sickle cell disease: Life expectancy and risk factors for early death. N. Engl. J. Med..

[28] Steinberg M.H., Lu Z.H., Barton F.B., Terrin M.L., Charache S., Dover G.J. (1997). Fetal hemoglobin in sickle cell anemia: Determinants of response to hydroxyurea. Multicenter Study of Hydroxyurea. Blood.

[29] Akinsheye I. (2011). Fetal hemoglobin in sickle cell anemia. Blood.

[30] Rigano P., De Franceschi L., Sainati L., Piga A., Piel F.B., Cappellini M.D., Fidone C., Masera N., Palazzi G., Gianesin B. (2018). Italian Multicenter Study of Hydroxyurea in Sickle Cell Anemia Investigators Real-life experience with hydroxyurea in sickle cell disease: A multicenter study in a cohort of patients with heterogeneous descent. Blood Cells Mol. Dis..

[31] Steinberg M.H., Barton F., Castro O., Ballas S.K., Armstrong F.D., Smith W., Ataga K., Swerdlow P., Kutlar A., DeCastro L. (2003). Effect of hydroxyurea on mortality and morbidity in adult sickle cell anemia: Risks and benefits up to 9 years of treatment. JAMA.

[32] Steinberg M.H. (2005). Predicting clinical severity in sickle cell anemia. Br. J. Hematol..

[33] Mark T., Gladwin M.D., Vichinsky E. (2008). Pulmonary Complications of Sickle Cell Disease. N. Engl. J. Med..

[34] Castro O., Brambilla D.J., Thorington B., Reindorf C.A., Scott R.B., Gillette P., Vera J.C., Levy P.S. (1994). The acute chest syndrome in sickle cell disease: Incidence and risk factors. Blood.

