# Supplementary material for: Chronic Administration of Hydroxyurea (HU) Benefits Caucasian Patients with Sickle-Beta Thalassemia
Source: Int J Mol Sci. 2018 Feb 28;19(3):681. doi: 10.3390/ijms19030681 (PMC5877542; doi:10.3390/ijms19030681)
Supplement: Supplementary file 1 [file ijms-19-00681-s001.pdf]

Supplementary Materials:

Table S1. Sickle genotype/phenotype.

Variable	Total (*N* = 140)
Median Age (Years)	35 (0.4-61)
Gender	
Male	69 (49%)
Female	71 (51%)
Genotype	
betaS/betaS	25 (17.8%)
betaS/beta039	35 (25%)
betaS/IVSnt110	33 (23.6%)
betaS/IVSnt1	10 (7.2%)
betaS/δβ	4 (2.8%)
betas/INS1nt6	16 (11.5%)
betaS/IVS2nt745	5 (3.6%)
betaS/-87	1 (0.7%)
betaS/IVS1nt5	1 (0.7%)
betaS/lepore	1 (0.7%)
betaS/INV2nt1	3 (2.1%)
not available	6 (4.3%)
Sickle Phenotype	
HbSS	25 (17.9%)
HbS/β0 thal	54 (37.9%)
HbS/β+ thal	56 (40.7%)
HbS/δβ thal	4 (2.8%)
HbS/Lepore	1 (0.7%)

Table S2. Mean hematologic and organ function parameters.

	First Visit			Last Visit		
Variable	No HU (*N* = 50)	HU <15 mg/kg/day (*N* = 30)	HU ≥ 15 mg/kg/day (*N* = 60)	No HU (*N* = 50)	HU <15 mg/kg/day (*N* = 30)	HU ≥ 15 mg/kg/day (*N* = 60)
Median HU dose	-	13.5	15		11.05	16.9
White blood count (K/uL)	11.2	8.74 *	10.9	10.7	7.8 *	8.3 *
ANC (K/uL)	5.84	5.39	5.65	4.98	4.55	4.16
Hemoglobin (g/dL)	10	10.2	10	10.2	9.7	9.9
Hemoglobin F (%)	11.9 **	9.4 *	10.7 *	7.7	11.7 *	12.8 *
ALT (U/L)	34.9	25.6	31.9	35.8	26.2	35.2
Total bilirubin (mg/dL)	2.32	1.96	2.99	2.81	2.31	2.95 *
Direct bilirubin (mg/dL)	0.50	0.35	0.52	0.74	0.54	0.83
Ferritin (mcg/L)	792.4	453	574	937.5	410.4 *	595.1
TRV (m/s)	2.50	2.61	2.54	2.64	2.62	2.72
Creatinine (mg/dL)	0.73	0.67	0.60	0.84	0.64	0.57

* $p < 0.05$ compared to no HU group; ** includes 4 subjects with high HbF levels and 2 children aged 3 months.
